# Addressing emerging infectious disease threats--accomplishments and future plans.

**DOI:** 10.3201/eid0403.980304

**Published:** 1998

**Authors:** J M Hughes

**Affiliations:** Centers for Disease Control and Prevention, Atlanta, Georgia, USA.


### Addressing Emerging Infectious Disease Threats—Accomplishments and Future Plans

**James M. Hughes**
Centers for Disease Control and Prevention, Atlanta, Georgia, USA

In 1962, Sir McFarland Burnet wrote, "One can think of the middle of the 20th century as the end of one of the most important social revolutions in history—the virtual elimination of the infectious disease as a significant factor in social life" (1). This statement is at the core of many years of neglect of infectious diseases—it represents complacency with a capital "C," and we are now paying the price.

Infectious diseases, the leading cause of death worldwide (2) and the third leading cause of death in the United States, have returned with a vengeance (3). Between 1980 and 1992, infectious disease deaths increased by 58% (39% after age adjustment); the major contributors were HIV infection and AIDS, respiratory disease (primarily pneumonia), and bloodstream infection.

In 1994, the Institute of Medicine published Emerging Infections: Microbial Threats to Health in the United States (4). This report broadly defined as emerging "new, reemerging, or drug-resistant infections whose incidence in humans has increased within the past two decades or whose incidence threatens to increase in the near future." This report, which detailed the factors involved in emergence, reminds us that we live in a global village.

Spurred on by the Institute of Medicine's report and by outbreaks of *Escherichia coli* O157 (January 1993), cryptosporidiosis (April 1993), and hantavirus pulmonary syndrome (May 1993), the Centers for Disease Control and Prevention and its partners produced a strategic plan for addressing emerging infectious diseases (5). The plan focused on increasing surveillance and response capacity; addressing applied research priorities; strengthening prevention and control programs; and repairing the public health infrastructure at local, state, regional, national, and global levels. Incremental implementation of this plan is ongoing. An update plan will be published in the fall of 1998.

### Addressing Emerging Infections in the United States: Implementation of CDC's Plan

### Emerging Infections Programs

Seven Emerging Infections Programs have been established through cooperative agreement awards (California, Connecticut, Georgia, Maryland, Minnesota, New York, and Oregon). These programs share core projects on invasive bacterial and foodborne diseases. The California program is focused on the San Francisco Bay Area. Four of the seven programs also focus on identifying the causes of unexplained deaths and severe illnesses in previously healthy persons ages 1 to 49 years.

### Epidemiology and Laboratory Capacity Cooperative Agreements

Thirty awards established cooperative agreements with 28 states and two large cities (Los Angeles and New York) (Figure). Funds are used in different ways in different locales, but each recipient works toward strengthening infectious disease surveillance capacity and improving laboratory capacity and the reporting and analysis of infectious disease surveillance data. In addition, CDC has established three new

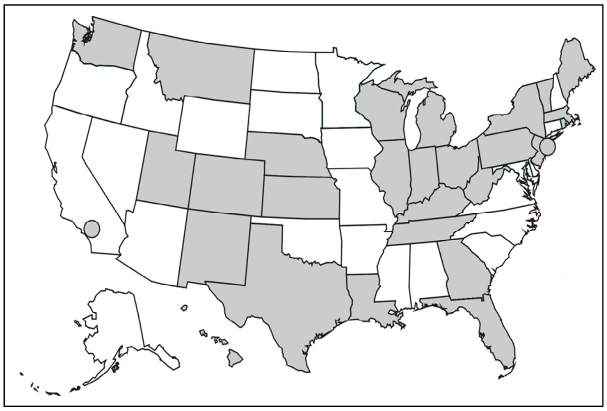

Figure. Epidemiology and laboratory capacity cooperative agreements (shown in gray).

provider-based sentinel surveillance systems with several partners. One network is based in emergency departments in academic medical centers (Emergency ID Network); a second, involving infectious disease clinicians, is in collaboration with the Infectious Diseases Society of America; and the third involves collaboration with the International Society of Travel Medicine (Geo-Sentinel), which involves travel medicine clinics in the United States and other countries.

### The National Food Safety Initiative

Because of inadequate foodborne disease surveillance in the United States, the safety of the food supply could not adequately be assessed. Six million to 81 million cases have been estimated (M. Osterholm, unpub. data). Food Safety from Farm to Table (6), released in 1997, underlines the Clinton Administration's commitment to improving food safety.

### The National Molecular Subtyping Network

The national molecular subtyping network (7) for foodborne disease surveillance (PulseNet) represents a model of disease surveillance that takes into account the globalization of the world's food supply. During the summer of 1997, the state public health laboratory in Colorado using molecular fingerprinting techniques (pulsed-field gel electrophoresis) recognized a cluster of 15 cases of *E. coli* O157:H7 infections from widely scattered areas in the state (8). Rapid epidemiologic investigation implicated undercooked ground beef from a single company, resulting in the recall of 25 million pounds of ground beef and the closing of the plant that produced it. This outbreak illustrates the critical role of public health laboratory capacity and rapid public health action in outbreak detection and response. Before the recent advances, this outbreak probably would not have been detected.

### The Emerging Infectious Diseases Laboratory Fellowship Program

In an effort to strengthen public health laboratory capacity, CDC in collaboration with the Association of State and Territorial Public Health Laboratory Directors will be providing opportunities for training state public health laboratory workers (9). Forty-five fellows have participated in this program. An international track will be inaugurated in the summer of 1998 with the support of the CDC Foundation and Eli Lilly and Company.

### The Emerging Infectious Diseases Journal

To better track trends and analyze new and reemerging infectious disease issues around the world, CDC established a quarterly, peer-reviewed international journal (www.cdc.gov/eid/). The journal, a part of the communications component of the strategy against emerging infections, has facilitated the exchange and dissemination of scientific information about these infections.

### Future Plans

Antimicrobial resistance, new and reemerging infections, and a strong public interest in health will demand vigilance, renewed efforts, and strengthened partnerships in infectious diseases. An update of CDC's strategic plan along with cooperative efforts across government and private organizations all over the world will drive future efforts for the control of new and reemerging infections.

### References

1. Burnet M, White DO. Natural history of infectious disease. London: Cambridge University Press; 1962.
2. World Health Organization. The World Health Report 1997: conquering suffering, enriching humanity. Report of the Director-General. Geneva, Switzerland: The Organization; 1997.
3. Pinner RW, Teutsch SM, Simonsen L, Klug LA, Graber JM, Clarke MJ, Berkelman RL. Trends in infectious diseases mortality in the United States. JAMA 1996;275:189-93.
4. Institute of Medicine. Emerging infections: microbial threats to health in the United States. Washington: National Academy Press; 1992.
5. Centers for Disease Control and Prevention. Addressing emerging infectious disease threats: a prevention strategy for the United States. Atlanta (GA): U.S. Department of Health and Human Services, Public Health Service; 1994.
6. U.S. Department of Health and Human Services, U.S. Department of Agriculture, U.S. Environmental Protection Agency. Food safety: from farm to table. A national food-safety initiative. A Report to the President, May 1997. Washington: Government Printing Office; 1997.
7. Stephenson J. New approaches for detecting and curtailing foodborne microbial infections. JAMA 1997;277:1337-40.
8. Centers for Disease Control and Prevention. *Escherichia coli* O157:H7 infections associated with eating a nationally distributed commercial brand of frozen ground beef patties and burgers—Colorado, 1997. MMWR Morb Mortal Wkly Rep 1997;46:777-8.
9. Emerging Infectious Diseases Fellowship Program. Emerg Infect Dis 1995;1:105.
